# Flood Detection in Gaofen-3 SAR Images via Fully Convolutional Networks

**DOI:** 10.3390/s18092915

**Published:** 2018-09-02

**Authors:** Wenchao Kang, Yuming Xiang, Feng Wang, Ling Wan, Hongjian You

**Affiliations:** 1 School of Electronic, Electrical and Communication Engineering, University of Chinese Academy of Sciences, Huairou District, Beijing 101408, China; xshzhdm@163.com (W.K.); wanling15@mails.ucas.ac.cn (L.W.); hjyou@mail.ie.ac.cn (H.Y.); 2 Institute of Electronics, Chinese Academy of Sciences, Beijing 100190, China; wfeng_gucas@126.com; 3 Key Laboratory of Technology in Geo-spatial Information Processing and Application System, Beijing 100190, China

**Keywords:** SAR, flood detection, FCN, GF-3 satellite

## Abstract

Emergency flood monitoring and rescue need to first detect flood areas. This paper provides a fast and novel flood detection method and applies it to Gaofen-3 SAR images. The fully convolutional network (FCN), a variant of VGG16, is utilized for flood mapping in this paper. Considering the requirement of flood detection, we fine-tune the model to get higher accuracy results with shorter training time and fewer training samples. Compared with state-of-the-art methods, our proposed algorithm not only gives robust and accurate detection results but also significantly reduces the detection time.

## 1. Introduction

Floods frequently occur from June to September in south and northeast China, causing a great deal of economic losses and disaster-induced diseases. According to the statistics from China National Commission for Disaster Reduction, there were 43 large-scale heavy rainfall events in 2017, which made 10 provinces in China suffer from heavy flood disasters. Consequently, it is necessary and urgent to cope with the flood disasters quickly. As the fundamental task of flood monitoring and rescue, flood detection needs to be accurate and fast.

With the rapid development of remote sensing sensors, many satellites have been launched, providing overall and continuous land cover information for disaster monitoring and damage assessment. Optical sensors, such as Landsat and SPOT, have been widely used for flood detection and inundation mapping. However, they are severely limited by meteorological conditions and cannot work at night due to their passive imaging characteristics. Unlike optical sensors, Synthetic Aperture Radar (SAR) sensors are active sensors and capable of working in all weather conditions and at all times of the day [1], which make emergency disaster monitoring through rain and clouds possible. Due to the coherent imaging mechanism and long revisit interval, SAR images are limited by speckle noise and data volume. Consequently, it is difficult but imperative to utilize SAR images for flood monitoring and rescue.

In the past decades, flood detection methods based on SAR images have been thoroughly studied. Some papers have made detailed summaries of these methods [1,2]. Flood detection methods can be mainly divided into four classes: thresholding, region growing, active counter, and hydrologic method.

Based on the assumption that flood SAR images have bimodal histograms, many automatic thresholding procedures are proposed [3,4]. Thresholding is the most classical method to distinguish flooded and non-flooded areas in SAR images. In thresholding method, pixels with radar backscatter values lower than a given threshold are assigned to the class “flood” [5,6]. Owing to relatively low computational cost, thresholding methods can meet real-time needs. However, since the decision threshold is affected by many factors such as atmospheric disturbances [7] and polarization mode [4], it has to be fine tuned by manual trial-and-error operations for each image. However, this assumption is not always true. Thresholding methods face a fundamental problem that not all pixels with low backscatter values are water and vice versa. Pulvirenti team presented some methods to overcome this problem [8,9,10]. One is to use change detection with InSAR data to deal with flood mapping in vegetated and urban areas [8]. Another way is a fuzzy logic method solving the interference of heavy rain or wet snow cover. Segmentation and classification are carried out successively with necessary ancillary data consisting of DEM, land cover map and optical image [9,10]. Although these methods are effective, the necessary auxiliary data are not always available.

Region growing is an iterative clustering method based on seed pixels. Adjacent pixels which have similar features with seed pixels are selected as new seed pixels. This process iterates until the stopping conditions are reached. Then, all these seed pixels are classified as water. There are two ways to initialize seed pixel or group seed pixels. One way used in practical engineering is to select seed pixels manually which is accurate but time-consuming, especially when flood regions are broken discontinuity. To solve this problem, another way is proposed to select seed pixels automatically. In these automatic methods, thresholding methods are used to give a threshold of seed pixels [11,12,13]. Therefore, these methods still suffer from the problems of thresholding methods.

Active contour model maps flood area by constructing an energy function and searching the minimum. By minimizing the energy function, the contour curve gradually approaches to the edge of interested object, and finally the target is segmented. For flood detection, the most commonly used active contour model is snake, an edge based model [14]. Although snake is useful to smooth edges and make them continuous, it can only process boundaries that have been defined by a gradient [15]. Another usual active contour model is level set method, a modified method of snake. Both snake and level set methods need a manual selection of the initial curves [16].

Hydrologic methods are also extensively used for flood mapping. All these methods use auxiliary data such as digital elevation model (DEM), hydrographic data, or topography information [2,17,18,19]. To achieve a better result, auxiliary data need to be high-precision and even be obtained during the flood [20]. It is difficult to acquire these data in time especially, when the informations is area oriented.

Although these methods have achieved some valuable results, even several automatic methods, they still have some limitations. First, these methods are sensitive to speckle noise, so they need to filter images with pre-processing. However, all filters now available will cause information loss. Besides, these methods are time consuming. To apply in emergency rescue, accurate results and real-time processing are both important. In addition, hydrologic method need extra auxiliary data, such as high accuracy DEM and water level information to get a better result, but these additional data are not always available.

In recent years, computer vision methods based on deep learning networks have developed rapidly and made great progress in image segmentation. At ILSVRC 2015, the best one, ResNet, achieved 3.57% error on the ImageNet test set, better than human eye recognition with 5.1% error [21]. Neural network has been used in SAR domain, such as ship detection [21], road extraction [22], and land cover classification [23], and achieved good results. However, we have not found any studies focused on deep-learning based flood detection methods in SAR images.

In this paper, we introduce a fully convolutional network (FCN) for flood detection which is based on classical deep learning method VGG16. FCN retains convolution layers and pooling layers in VGG16 to extract features, but replaces fully connected layers with transpose convolution layers to map flood areas. Our method is robust to speckle noise with no need for filtering. Furthermore, FCN can process single image of arbitrary polarization without auxiliary data, so it has good generalization. The contributions of this paper are given as follows:

1. To the best of our knowledge, this is the first paper to introduce fully convolution network (FCN) for flood detection in SAR images.
2. We verified that FCN is robust to speckle noise in SAR image.
3. For the needs of SAR image flood detection, we fine-tuned the classical FCN in three points.

The rest of this paper is organized as follows. The study areas and data used in this paper are described in Section 2. Section 3 contains three parts, the pre-processing of data, the method for making the dataset and the flood mapping method. We illustrate and analysis experimental results in Section 4. The conclusions are drawn in Section 5.

## 2. Study Area

According to the information from China National Commission for Disaster Reduction, there are five heavy floods in China among the ten worst natural disasters in 2017. Floods and geological disasters in 2017 influenced 69.512 million people in China, among which 674 people died, 75 people are missing and 3.975 million people were resettled in an emergency. These disasters caused a total direct economic loss of 190.99 billion yuan.

Gaofen-3 satellite (GF-3) is China’s first self-developed C-band multi-polarized SAR, launched in August 2016. GF-3 can obtain full-polarization, dual-polarization and single-polarization images. Gf-3 has twelve imaging modes with 1–500 m resolution and 5–650 km imaging bandwidth. The design life of GF-3 is eight years. All these characters make GF-3 competent for numerous applications such as monitoring the global ocean and land resources [24]. Flood mapping is one of the import applications. We chose three scenes of GF-3 images from three different flood disasters in 2017 China for our study. These images were imaged in three different modes.

**Figure 1:**
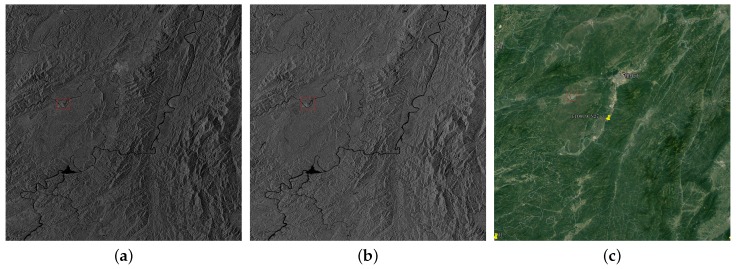

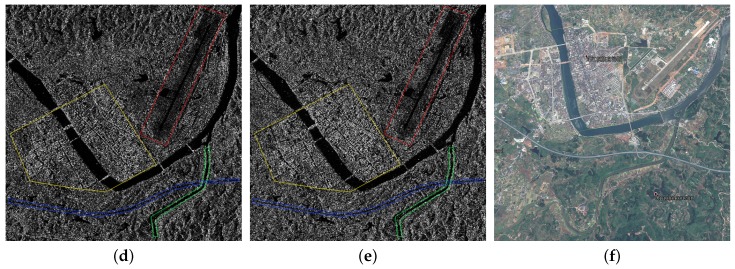
(**a**) HH; and (**b**) HV are dual polarization GF-3 images of Hunan province (3 July 2017) imaged in FSII mode with 10 m resolution covering 10,894.8 square kilometers. Image size is 10,996 × 9908 pixels. (**c**) The optical image of the same area obtained from Google Earth. (**d**–**f**) Selected areas of (**a**–**c**). City, airport, highway, and part of water are marked by yellow, red, blue and green frames, respectively.

Scene 1 was imaged in fine stripmap 2 (FSII) mode on July 3 in dual polarization with 10 m resolution covering 10,894.8 square kilometers. This scene is about the flood in Hunan province caused by continuous heavy rainfall. The image center is located in (E109.9, N27.3). The two images of HH and HV are shown in Figure 1a,b. We also present the optical image obtained from Google Earth as a reference in Figure 1c. Except water, the land cover classifications also contain mountain forests, croplands, cities and small towns. Figure 1d–f shows the selected parts in Figure 1a–c. City, airport, highway and part of water are marked by yellow, red, blue and green frames, respectively. Limited by the image resolution, the inner structure of city is impossible to distinguish. Thus, we do not consider the influence of street and building shadows in city. Airport and highway have similar reflectivity with water which may cause false alarms.

**Figure 2:**
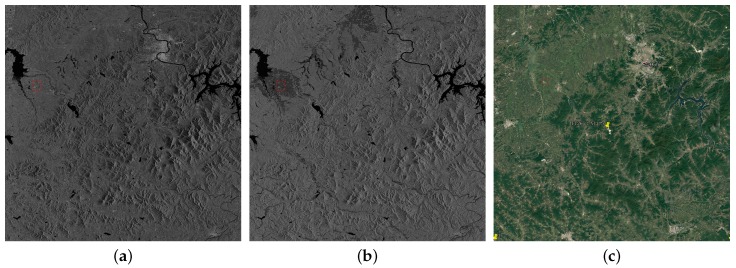

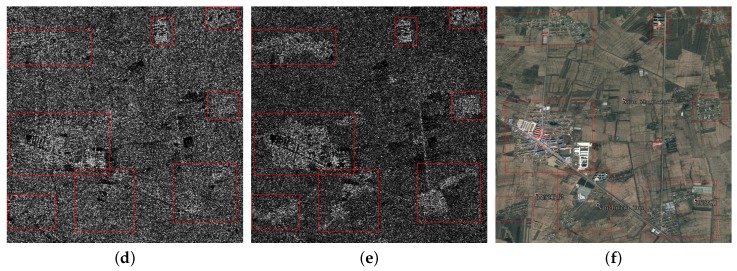
(**a**) HH; and (**b**) HV are full polarization GF-3 images of Jilin province (13 July 2017) imaged in SS mode with 25 m resolution covering 83,359.2 square kilometers. Image size is 10,953 × 12,177 pixels. (**c**) The optical image of the same area obtained from Google Earth. (**d**–**f**) Selected areas of (**a**–**c**). Areas in red mark are buildings and the rest is cropland.

Scene 2 was imaged in scan SAR (SS) mode on July 13 in dual polarization with 25 m resolution covering 83,359.2 square kilometers. This scene is about the flood in Jilin province. The image center is located in (E126.3, N43.5). The two images of HH and HV are shown in Figure 2a,b. We also present the optical image obtained from Google Earth as a reference in Figure 2c. The land cover classifications are the same as Scene 1. We can see evident difference between HH and HV polarization in Figure 2a,b. We select one of the most obvious areas to show in Figure 2d–f. Main building areas are marked with red frames and the rest is cropland. The building areas in HH polarization image are almost the same as croplands while they have obvious different intensities in HV polarization image.

**Figure 3:**
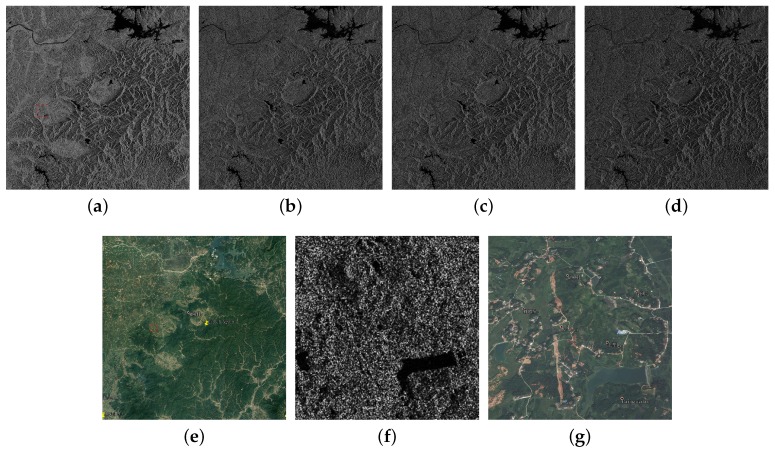
(**a**) HH; (**b**) HV; (**c**) VH; and (**d**) VV are fully polarimetric GF-3 images of YueYang city in Hunan province (17 August 2017) imaged in QPSI mode with 8 m resolution covering 884.7 square kilometers. Image size is 3859 × 3582 pixels. (**e**) The optical image of the same area obtained from Google Earth. (**f**,**g**) Selected areas of (**a**,**e**). Buildings are fused with other classifications.

Scene 3 was imaged in Quadrupolarization Stripmap One (QPSI) mode on August 17 in full polarization with 8 m resolution covering 884.7 square kilometers. This scene is about the flood in YueYang city, the second heavy flood in Hunan in 2017. The image center is located in (E113.5, N29.0). The four images of HH, HV, VH and VV are shown in Figure 3a–d. The reference optical image is shown in Figure 3e. Different from Scenes 1 and 2, there is no big city in Scene 3. Although Scene 3 has the highest resolution, buildings in small towns still cannot be recognized, as shown in Figure 3f,g.

## 3. Methodology

In this section, we introduce the pre-processing for making the datasets, the selecting method for the datasets and the structure of our model. Pre-processing plays an important role in our model to acquire a good result. It contains two procedures, radiation correction and data unification. We give a new selecting method for datasets in Section 3.2. In the last section, we introduce each part of our model in detail.

### 3.1. Pre-Process

In general, the GF-3 data are Level-1 product (L1A), i.e. 16-bit complex data that cannot be used for flood mapping directly. We should convert the complex data to digital number (DN). The intensity image is obtained by:

(1) $$DN=\sqrt{I^{2}+Q^{2}},$$

where *I* and *Q*, respectively, represent the real part and virtual part of L1A data. The intensity image has a problem that the water area of DN is different in different scenes. Thus, we need to perform radiation correction on L1A data and convert it into a figure of ground scattering coefficient $\sigma^{0}$ by:

(2) $$\sigma^{0}=\frac{{\left(\frac{I}{32,767}*Qualify\right)}^{2}+{\left(\frac{Q}{32,767}*Qualify\right)}^{2}}{K_{const}},$$

where Qualify is the Qualify Value which can be found in xml document of L1A data and $K_{const}$ is the calibration constant which is get by radiometric calibration. $K_{const}$ is different for different imaging model, different band and different polarization.

After radiation correction, we need to scale the σ map to 0:65,535. Due to the existence of speckle noise, directly using linear scaling is not suitable for useful ground scattering coefficients, being compressed to a very small range. In this way, we can only get almost black images. To solve this problem, we set a threshold. σ values greater than the threshold are set to the threshold. We choose the threshold by ensuring more than 90% of values are lower than the threshold. Although some effective σ values are abandoned, flood detection is not influenced because flood pixels have low σ values. The same ground feature has a lower σ value in cross-polarization than in co-polarization. Thus, we choose the threshold depending on polarization to unify data. After experiments, we found some feasible thresholds, as given in Table 1, with which different polarization images have similar σ values for same classification. This is useful for model training. Finding the most universal threshold requires analyzing more data and we will do this in future.

Due to coherent imaging mechanism, SAR images are limited by speckle noise. However, we do not use filtering. As we all know, adding noise is a common data augmentation skill for deep learning training sample making to make model more universal. Thus, speckle noise is similar to data augmentation for SAR image. In addition, filtering is bound to cause loss of details in images and bring extra computational cost. Thus, we train our model directly without filtering. This completes the pre-processing explanation. We verify the effectiveness of pre-processing and filtering in Section 4.4.

### 3.2. Dataset Making

Now, we have eight original images (relative to slice set) to make datasets. For training set and validation set, we use Scenes 1 and 2 and keep the same proportion of positive samples (samples having water pixels) and negative samples (samples having no water pixels) in both sets. We slice these four images into patches with a size of 256 × 256 pixels and a stride of 192 pixels. We randomly select a certain percentage (marked as p) of positive samples with the same amount of negative samples as training set. The remaining positive samples with the same amount of negative samples serve as validation set. We use one image in Scene 2 as instance. By slicing, we can get$\left\lfloor (10,953-256)/192+1\right\rfloor *\left\lfloor (12,177-256)/192+1\right\rfloor =3528$ samples containing 1136 positive samples and 2392 negative samples. In Section 4.2, we select p=80%. Then, we randomly select 1136∗p=908 samples from positive samples and 908 samples from negative samples. We use these 1816 samples as training set. In the same way, we use the rest 1136−908=228 positive samples and 228 negative samples randomly selected from the rest 1484 negative samples as validation set (total 456 samples).

For the test set, we have found that test sets in previous papers are small areas with only a few hundred to a few thousand pixels [11,12,15,20]. Besides, the part of test set is captured in the same image with training set. We think there are three problems with these test sets.

1. The selected part of test set is too small. As SAR images resolution becomes higher and higher, only thousands of pixels cannot cover a large complete water area, while the 256 × 256 pixels patch used in our training set has more than 60 thousand pixels.
2. Selecting a particular part as test set has a great randomness. The purpose of training is to map the flood area in whole image. However, the result on a small area is difficult to evaluate the effect of the model on the whole image.
3. These test sets cannot fully reflect the generalization ability of model. Since the test set is captured in the same image with the training set, we have no way to know the model performance on other scenes. That is exactly the most important objective of our training model.

To overcome the aforementioned problems, we propose a new test set selection method for supervised learning method. We test the model performance on the four original images in Scenes 1 and 2 to avoid the first and second problems. In addition, we make a test on Scene 3, a new group of data never participating in training, to validate the generalization ability of model. Moreover, we want to know whether our model has a similar performance on different polarization images after the pre-process.

### 3.3. FCN

Traditional network can only process images having the same size with training samples limited by the fixed numbers of parameters in fully connected layers. In common, neural networks choose small sizes (e.g., 256 × 256 pixels) training samples to improve training effect. Thus, traditional networks are inefficient when process large size images. Fully convolutional networks (FCN) replace fully connected layers in traditional neural networks with convolutions. Thus, one advantage of FCN is that it can handle images of any size after it has been trained, which improves the efficiency of network.

**Figure 4:**
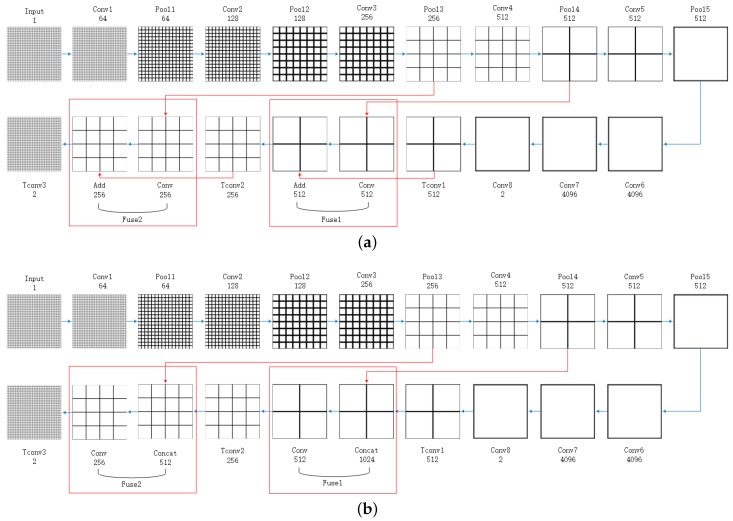
The training processes of the two models: (**a**) FCN; and (**b**) FCN16. The number of grids reveals relative spatial coarseness. Convolution numbers of ConvX are given in Table 2. Both models upsample stride 32 predictions back to pixels in three steps. Tconv1 and Tconv2 achieve double upsampling and Tconv3 achieves eight times upsampling. Different structures between the two models are marked with red. The number under layer name is channel number.

Shellamer et al. [25] introduced a FCN model for semantic segmentation on optical images that is based on the structure of classical network VGG16 [26]. This network contains two parts: convolution layers which are same with VGG16 and transpose convolution layers which are substitutes of fully connected layers in VGG16. To make a distinction with our model, we still call this model FCN. Based on FCN, we propose a fully convolutional network for flood detection on SAR images, which we called it FCN16. The structures of FCN and FCN16 are compared in Table 2. We also show the training processes of FCN and FCN16 in Figure 4. The selection bases of the layers are given in follow.

**Convolution layer:** Traditional networks use big size kernels to get large receptive fields. As we all know, the smallest kernel size is 3 × 3 to capture the information of a pixel and its surrounding pixels. Karen Simonyan et al gave an opinion that a stack of 3 × 3 kernel can get the same size receptive fields with big size kernels with fewer parameters [26]. For example, a single 7 × 7 layer (1 × 7 × 7 = 49 parameters) can be replaced by a stack of three 3 × 3 layers (3 × 3 × 3 = 27 parameters) with the receptive fields 7 × 7 unchanged. Another advantage is that a stack structure has two more activation functions which improve the expression ability of the model. Thus, we keep this structure in FCN16. Moreover, we use rectified Linear Unit (ReLU) activation function for every convolution layer except conv8. Actually, we have tried other improved variants of ReLU, such as ELUs [27] and SELU [28], but the results had no improvement or were even worse.

The difference between FCN and FCN16 is in conv6, where FCN16 uses 3 × 3 kernels instead of 7 × 7 kernels in FCN. In the original VGG16 network, the transition from convolution layer to fully connected layer can be treated as a 7 × 7 convolution, so FCN network replaces this transition with a 7 × 7 convolution layer in conv6. We replace these 7 × 7 kernels with 3 × 3 kernels in our model for two reasons. First, although a big kernel can increase the number of features, it also makes the model more complex and difficult to train for tending to be overfitting. Considering that flood detection is only a binary classification problem and we want to get a model which can be trained with fewer samples and shorter time, the model should not be too complex, so we replace 7 × 7 kernels with 3 × 3 kernels. In this way, the model parameters in conv6 reduce from 108 M to 19 M and this will greatly speed up the model training. Second, the single node in the first fully connected layer has the information of all pixels in the original image, i.e., the receptive field is bigger than the sample size. For this purpose, a 3 × 3 kernel with a 276 × 276 pixels receptive field is sufficient for 256 × 256 pixels training samples and a 7 × 7 kernel with a 404 × 404 pixels receptive field cannot give more information. Thus, we use 3 × 3 kernels replace 7 × 7 kernels.

**Pooling layer:** Pooling layer, commonly maximum pooling, is used to reduce the dimension of data. For this purpose, we can remove pooling layer and add the stride of former convolution layer to 2. In this way, network training time can be further reduced. However, Springenberg et al. indicated that pooling layer is unnecessary but simply removing pooling layer leads to a poor performance of classification, because only top-left feature is considered [29]. In other words, max-pooling uses top-left feature as default. Thus, we keep the pooling layers and compare two methods in subsequent section.

**Dropout layer:** Dropout layer can suppress overfit to some extent which was demonstrated by Srivastava N [30] and Xavier Bouthillier [31] from different arguments. We add dropout layers after the conv7 and conv8. The dropout rate is fixed as 0.5. Since overfitting is not a serious problem in our experiment, the effect of dropout layers is limited.

**Transpose convolution layer:** Transpose convolution (also called deconvolution in some papers) is used to upsample images, playing a crucial role in FCN. Shellamer et al. demonstrated that using three transpose convolution layers to upsample final convolution result to original size was the most effective way [25]. We also verified this conclusion by changing the number of transpose convolution layers and the size of kernel.

**Fusion layer:** FCN uses skip structures (e.g., U-net) to fuse local features from shallower layers with global features from deeper layers. We call this structure fusion layer. In Figure 4a, we can see that fusion layers in FCN contain two steps. First, it uses a 1 × 1 convolution to further extract features from the shallower layer (pool4 or pool3). Then, it adds this result with the deeper layer (tconv1 or tconv2). This is similar to adding an exclusive bias for each pixel in these new features, although this bias depends on the deeper layer. We think simply using global features as biases in the corresponding pixel cannot efficiently utilize global features, so we incorporate the deeper layers into convolution process in FCN16. As shown in Figure 4b, we still use a 1 × 1 convolution, but the input of convolution is the concatenation of the shallow layer and deep layer. In this way, we extract new features using both local features and global features. FCN can be seen as a particular case that the convolution weights corresponding to deeper layer are fixed to (1, 0, 0, ..., 0, 0), (0, 1, 0, ..., 0, 0), ... , (0, 0, 0, ..., 0, 1). Thus, our model has a better expressive ability. In addition, we add a ReLU function after convolution to further improve the expressive ability of the model.

**Loss function:** We only use cross entropy loss function, a commonly used loss function in classification task. Except this function, FCN also uses L2 regular function (regular factor is $5\times 10^{-4}$) as part of total loss function. L2 regular function is usually used to overcome overfitting. L2 regular function suppresses overfitting by reducing weights to simplify the model, which is not suitable for our use because overfitting is not a serious problem in flood detection, especially after we simplified the model by reducing the feature amount. Thus, we replace the L2 regular function with dropout layers to prevent possible overfitting. Actually, adding L2 regular function will make result worse instead. We show this in Section 4.4.

## 4. Results and Discussions

Four experiments were designed to evaluate the detection performance. In the first experiment, the proposed detection method was compared with FCN and a traditional automatic method M1. In the second experiment, the influence of training sample numbers was studied. In the third experiment, we analyzed the effectiveness of pre-process and L2 regular function. In the last experiment, we explored the performance of removing the pooling layer.

### 4.1. Assessment Criteria

Confusion matrix or criteria based on it are the most commonly used tools for evaluating flood detection ability [12,15,32]. We chose four criteria based on confusion matrix. The confusion matrix used in this paper is given in Table 3. T is true, F is false, P is positive (i.e., regarding pixel as water) and N is negative (i.e., regarding pixel as other classification). The four criteria are accuracy (A), recall (R), precision (P), and F1 score (F1). The mathematical formulation are:

(3) $$\begin{array}{c} A=\frac{TP+TN}{TP+FN+FP+TN},\\ R=\frac{TP}{TP+FN},\\ p=\frac{TP}{TP+Fp},\\ F1=\frac{2*R*P}{R+P}. \end{array}$$

where A represents the proportion of correctly classified pixels in the total number of pixels. R represents how many water pixels are correctly detected. P represents the correct proportion of pixels classified as water. The best effect of model is both R and P are large, but in fact, we always need to make a balance between these two criteria. Thus, we use criterion F1 to measure this balance.

### 4.2. Comparison of Three Methods

In this section, we compare our model with two methods. One is the FCN shown in Table 2. Another model is a state-of-the-art automatic flood mapping method called M1 [11]. We chose M1 because this method only uses one image to detect floods which is the same as our method. Although Matgen et al. [11] introduced another method M2, which performs better than M1, and there exist some methods that further improve M2 (e.g., [12,13]), all these methods need an extra image (i.e., multi-temporal data) to obtain the result. M1 is a hybrid method combining thresholding and region growing based on the assumption that flood images have binodal histograms. However, influenced by speckle noise, the original SAR images do not have binodal histograms. Thus, Gauss filter is used for M1 to generate bimodal histograms. Based on bimodal histogram, M1 uses thresholding to generate seed pixels for region growing automatically. Then, region growing is used to map all water areas in image.

**Figure 5:**
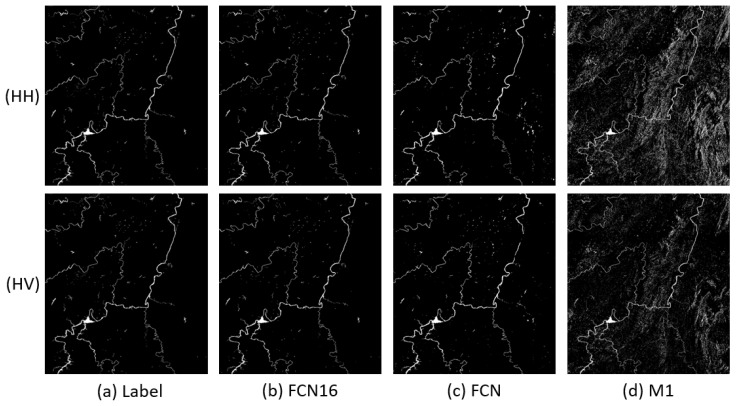
Results comparison of Scene 1: (**a**) Label; (**b**) FCN16; (**c**) FCN; and (**d**) M1.

**Figure 6:**
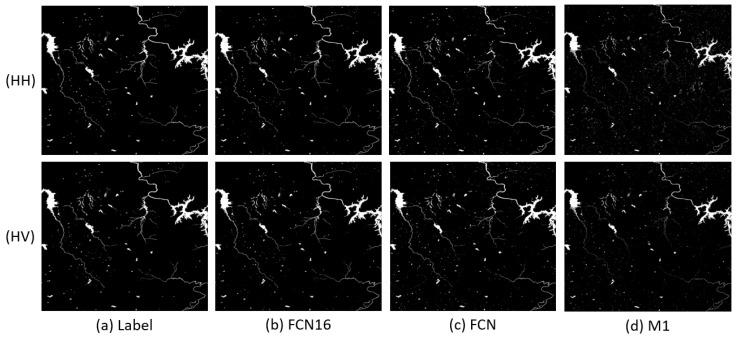
Results comparison of Scene 2: (**a**) Label; (**b**) FCN16; (**c**) FCN; and (**d**) M1.

**Figure 7:**
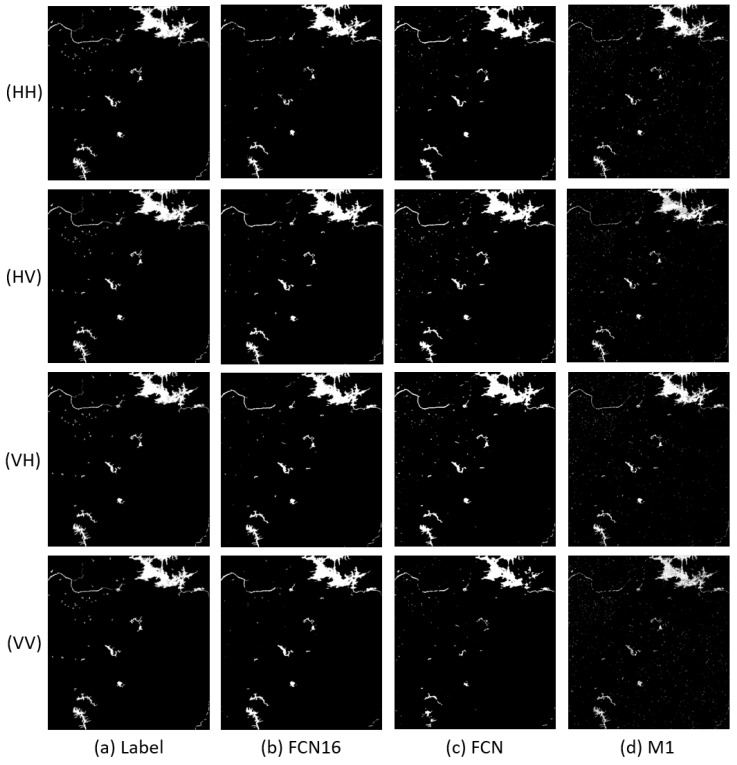
Results comparison of Scene 3: (**a**) Label; (**b**) FCN16; (**c**) FCN; and (**d**) M1.

In this section, p is set to 80% to ensure there are sufficient training samples to get a satisfactory result. Learning rate and iteration are, respectively, fixed as $10^{-4}$ and 30 k in this and the following section for both FCN and FCN16. The results of Scenes 1–3 are shown in Figure 5, Figure 6 and Figure 7, respectively. The first column (Label) in each figure is the real flood map manually marked.

We can see that the FCN16 gives the best results and M1 is the worst, especially in Scene 1. For all methods, performances on HH, HV, and VH are better than the performance on VV. A possible reason is that VV polarization provides less information for the application of flood mapping than other polarizations [4].

In Scene 1, the flood images of FCN has more errors in lower right corner than FCN16. We can see in original images that this part is a mountainous area with shadow. The results of M1 is much worse than FCN16. It seems that M1 is not robust to mountain shadow. In Scene 2, we can see more messy points caused by small shadow areas in FCN and M1 than FCN16. It is obvious that the results of M1 are still the worst. In Scene 3, the results are similar with Scene 2. We can also find that M1 is sensitive to speckle noise, although Gauss filter has been used. The noise points in top right corner of the large flood area are all kept in flood map of M1. FCN16 and FCN overcome this problem.

To clearly analyze the effectiveness of FCN16, we compared the quantitative indices in Table 4. FCN16 acquires the best results in almost all indices for every image. For A, all values of FCN16 are more than 0.99; 0.0015–0.0058 more than FCN and 0.0029–0.0871 more than M1. The improvement of A is not very remarkable if we ignore the results of Scene 1, after all the values of A are very close to 1. For F1, all values except the VV in Scene 3 of FCN16 are more than 0.9; 0.0308–0.1550 more than FCN and 0.0646–0.6631 more than M1. FCN16 makes a great progress in F1, which means FCN16 achieves a good balance between R and P while improving both criteria.

Moreover, we compare the training time and test time in Table 5. We trained and tested FCN16 and FCN on a single NVIDIA GeForce GTX 1080Ti GPU. Compared with FCN, we reduced training time by more than half and only needed 1 h 8 min to train a new model. As for test time, processing eight images with FCN16 only needed about 2 min while M1 needed 30 min. Thus, a pre-trained FCN16 is more in line with real-time processing requirements. In summary, FCN16 has more practicability than the other two methods.

### 4.3. Analyses of Training Sample Numbers

There is a common problem with all supervised training models on FCN16 that these models cannot process new data with significant characteristic differences. The only way to solve this problem is to use new data to train a new model. However, marking label manually is a time consuming task. It will be a large progress if we can train model with as few training samples as possible on a premise of obtaining a satisfactory result. In Section 4.2, we have achieved a good result with 80% positive patches contained in training set and compared the superiority of FCN16 over FCN and M1. We tested the efficiency of FCN16 under different training sample numbers. In other words, we tested how many training samples are necessary to acquire a acceptable result.

**Figure 8:**
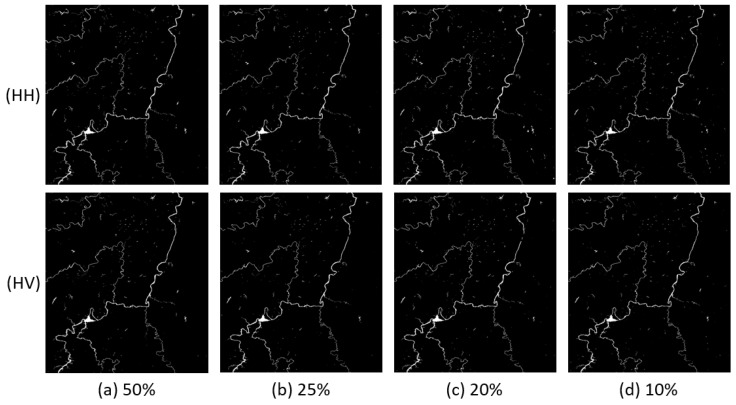
Results comparison of Scene 1 with different p: (**a**) p = 50%; (**b**) p = 25%; (**c**) p = 20%; and (**d**) p = 10%.

**Figure 9:**
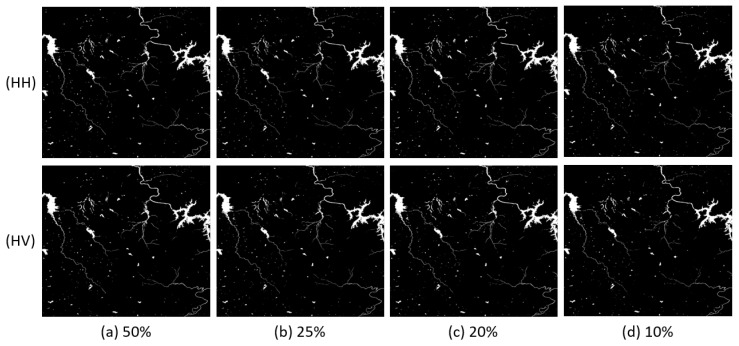
Results comparison of Scene 2 with different p: (**a**) p = 50%; (**b**) p = 25%; (**c**) p = 20%; and(**d**) p = 10%.

**Figure 10:**
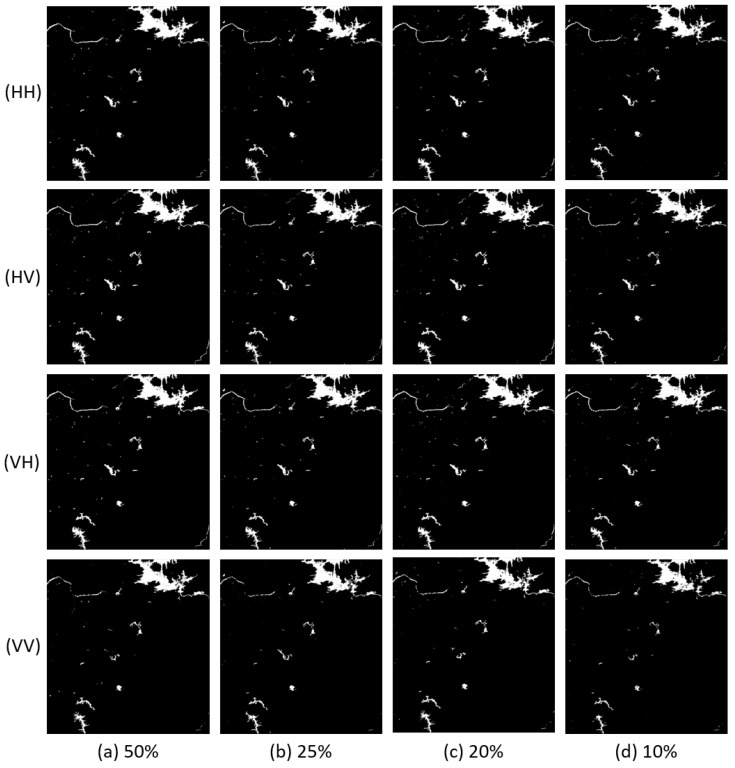
Results comparison of Scene 3 with different p: (**a**) p = 50%; (**b**) p = 25%; (**c**) p = 20%; and (**d**) p = 10%.

In this experiment, we changed parameter p to 50%, 25%, 20% and 10%, respectively, to train FCN16. We list the results of flood maps in Figure 8, Figure 9 and Figure 10. The difference among each image is not very obvious in view.

**Figure 11:**
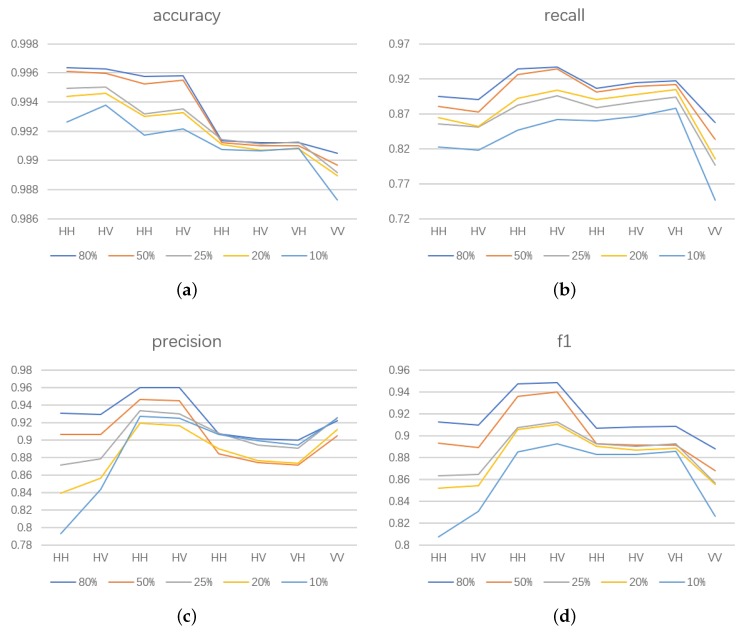
The comparison of four indices under different training set sizes: (**a**) accuracy; (**b**) recall; (**c**) precision; and (**d**) F1 score. In each figure, the horizontal axis represents different images and the vertical axis represents the corresponding index.

The comparisons of the indices are shown in Figure 11. We can see that the results of Scene 3 are more stable than Scenes 1 and 2. This is easy to understand ,as complicated images need more training samples to learn enough features.

Although the indices of FCN16 decrease as the training samples decrease, even the worst result with 10% positive samples is still better than the result of M1. To see this clearly, we list the results of these two experiments in Table 6. Except the precision of HH, HV, and VH in Scene 3, all indices of FCN16 are better. Compared with FCN16, M1 sacrifices more recall to get a better precision. This is verified by the F1 scores. Thus, we can conclude that FCN16 has potential to achieve satisfactory results with fewer training samples. If there is only one image to map flood area with a new model, we recommend that the positive patches in training set are better than 20% of all flood areas in image.

### 4.4. Analyses of the Pre-Process and L2 Regualr Fuction

**Figure 12:**
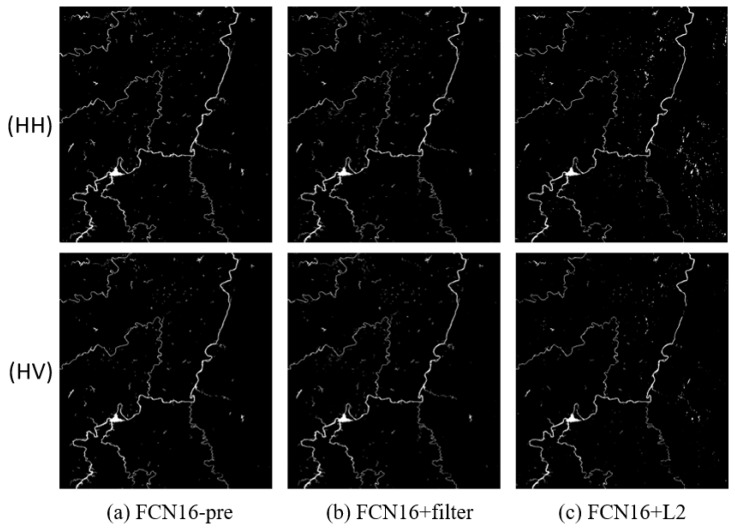
The results of FCN16 in three different conditions of Scene 1: (**a**) FCN16 without pre-process; (**b**) FCN16 with filtering after pre-process; and (**c**) FCN16 using L2 regular in loss function.

**Figure 13:**
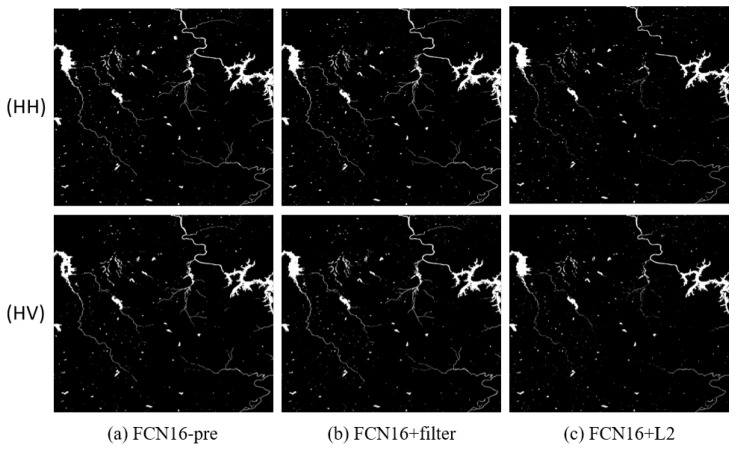
The results of FCN16 in three different conditions of Scene 2: (**a**) FCN16 without pre-process; (**b**) FCN16 with filtering after pre-process; and (**c**) FCN16 using L2 regular in loss function.

**Figure 14:**
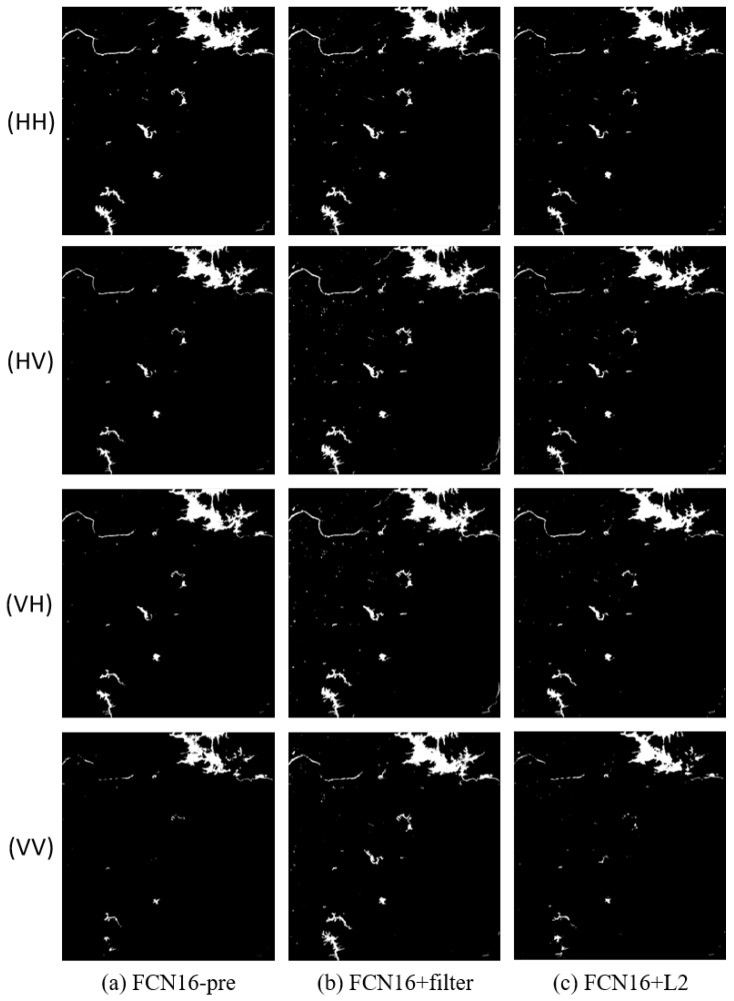
The results of FCN16 in three different conditions of Scene 3: (**a**) FCN16 without pre-process; (**b**) FCN16 with filtering after pre-process; and (**c**) FCN16 using L2 regular in loss function.

We compared the results of FCN16 in three different conditions. The first used data that were not pre-processed for training to verify the effectiveness of pre-processing, marked as FCN16-pre. The second added filtering in pre-processing to verify its necessity, marked as FCN16+filter. The third added L2 regular function in loss function to verify the conclusion in Section 4.3 that using L2 regular makes result worse, marked as FCN16+L2. Thr parameters are the same as in Section 4.2. The results are shown in Figure 12, Figure 13 and Figure 14 and Table 7.

In Table 7, nearly all indices of FCN16-pre are worse than FCN16 with a clear gap, which means pre-processing plays an important role in improving the result.

Although recall of FCN16+filter is better than FCN16, this comes at the expense of accuracy. The worse F1 score indicates that this interchange is inadvisable. A possible reason is that filtering leads to the loss of details. Thus, we do not recommend filtering. This contrast experimental result can also be a useful reference for the subsequent segmentation and extraction of SAR image using FCN.

**Figure 15:**
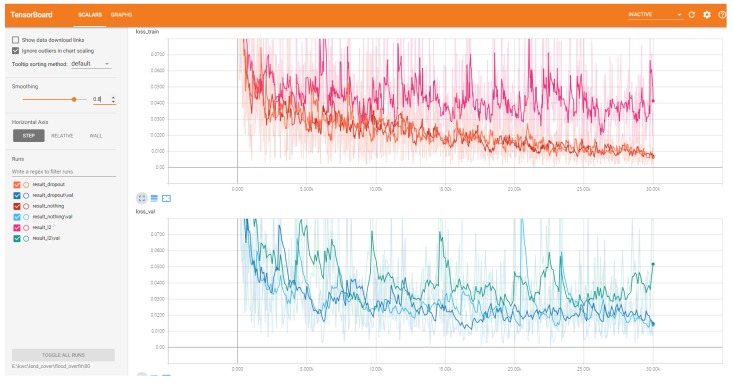
The loss value curves of training samples and validation samples during the training period in three cases: using dropout (i.e., FCN16), using L2 regular function and using nothing (reduce dropout in FCN16).

For FCN + L2, nearly all indices are worse than FCN16. As we have mentioned, this is because L2 regular function limits the model expression ability. To see this clearly, we compared the loss values of training samples and validation samples during the training period in three cases: using dropout (i.e., FCN16), using L2 regular function and using nothing (reduce dropout in FCN16). We show this in Figure 15. Compared to the other two cases, the curve using L2 regularization is maintained at a large loss, i.e., the model expression ability is limited. Thus, we do not use the L2 regular function in our model.

### 4.5. Analyses of the Necessity of Pooling Layer

In this experiment, we wanted to see if we could further improve the efficiency of network training. Without changing the number of network parameters, the only way we could consider is to remove the pooling layers. As mentioned in Section 3.3, this change will make the result worse, but we wanted to know if the deterioration of the result is acceptable. Thus, we used 80% positive samples training the model with no pooling layers. All parameters are the same as FCN16 in Section 4.2.

Training time of the simplified model was 3791.373 s and test time was 108.3594 s. Compared with FCN16, the training time decreased 273.0354 s. We give the results of the simplified model and the difference Δ with FCN16 in Table 8. Positive Δ means reducing pooling layer is a better choice. We can see the decrement of recall values are much more than the increment of precision in Scenes 1 and 2. Nearly all indices in Scene 3 become worse. In other words, the simplified model has poor generalization ability. The deterioration is more seriously than the improvement when only a little training time is reduced. Thus, we recommend using the pooling layers.

## 5. Conclusions

This paper introduces a fully convolutional network FCN16 based on the classical FCN for flood mapping. For the needs of SAR image flood detection, we fine-tuned the classical FCN in three points: (1) We simplified the model by replacing 7 × 7 kernels with 3 × 3 kernels so it can be trained with fewer samples and shorter time. (2) To improve the model expression ability, we used dropout layers to prevent overfitting instead of the L2 regular function in classical FCN. (3) We changed the structure of fusion layers to improve the model expression ability. FCN16 was tested and evaluated against FCN and a traditional automatic algorithm M1 on three scenes. In this paper, the three scenes we used are imaged by three different modes of GF-3 which are usually used for disaster reduction. FCN16 makes a small improvement in accuracy of 0.0015–0.0058 more than FCN and 0.0029–0.0871 more than M1. This is because the accuracy is very close to 1 and even the worst one is more than 0.98. The important thing is that FCN16 makes very large improvement in F1 score of 0.0308–0.1550 more than FCN and 0.0646–0.6631 more than M1. This means that FCN16 has achieved excellent overall performance. Moreover, time used for processing images with a trained FCN16 is far less than M1. It is almost real-time processing. Besides, our model does not need to filter images, so we can keep as much original information as possible. We have also demonstrated that FCN16 was able to achieve a satisfactory result with only a short training time and few training samples. Thus, we can quickly train a new model to deal with new data that are quite different with existing data. In actual use, operators only need to manually mark a few samples, which greatly improves the work efficiency.

In our future work, we will consider improving the ability of model transfer learning to make the model more practical. Besides, inspired by some traditional methods, we will study the flood transit area mapping method based on deep learning networks in future work [33,34].

## Figures and Tables

**Table 1:** The thresholds of different polarization.

Polarization	HH	HV	VH	VV
Threshold	0.6	0.1	0.1	0.6

**Table 2:** Comparison of structures between FCN and FCN16 (The column Num in Layer is the number of convolutions in convolution layer).

Layer		FCN			FCN16		
Name	Num	Kernel Size	Stride	Feature Dimensions	Kernel Size	Stride	Feature Dimensions
input				256 × 256 × 1			256 × 256 × 1
conv1	2	3 × 3	1	256 × 256 × 64	3 × 3	1	256 × 256 × 64
pool1		2 × 2	2	128 × 128 × 64	2 × 2	2	128 × 128 × 64
conv2	2	3 × 3	1	128 × 128 × 128	3 × 3	1	128 × 128 × 128
pool2		2 × 2	2	64 × 64 × 128	2 × 2	2	64 × 64 × 128
conv3	3	3 × 3	1	64 × 64 × 256	3 × 3	1	64 × 64 × 256
pool3		2 × 2	2	32 × 32 × 256	2 × 2	2	32 × 32 × 256
conv4	3	3 × 3	1	32 × 32 × 512	3 × 3	1	32 × 32 × 512
pool4		2 × 2	2	16 × 16 × 512	2 × 2	2	16 × 16 × 512
conv5	3	3 × 3	1	16 × 16 × 512	3 × 3	1	16 × 16 × 512
pool5		2 × 2	2	8 × 8 × 512	2 × 2	2	8 × 8 × 512
conv6	1	7 × 7	1	8 × 8 × 4096	3 × 3	1	8 × 8 × 4096
dropout							8 × 8 × 4096
conv7	1	1 × 1	1	8 × 8 × 4096	1 × 1	1	8 × 8 × 4096
dropout							8 × 8 × 4096
conv8	1	1 × 1	1	8 × 8 × 2	1 × 1	1	8 × 8 × 2
tconv1		4 × 4	2	16 × 16 × 512	4 × 4	2	16 × 16 × 512
fuse1		1 × 1	1	16 × 16 × 512	1 × 1	1	16 × 16 × 512
tconv2		4 × 4	2	32 × 32 × 256	4 × 4	2	32 × 32 × 256
fuse2		1 × 1	1	32 × 32 × 256	1 × 1	1	32 × 32 × 256
tconv3		16 × 16	8	256 × 256 × 2	16 × 16	8	256 × 256 × 2

**Table 3:** Confusion matrix.

Confusion Matrix		Prediction	
		Water	Other
Truth	Water	TP	FN
	Other	FP	TN

**Table 4:** The indices of three different methods. A is accuracy, R is recall, P is precision, and F1 is F1 score, given in Equation (3). The best results are in bold.

		Scene 1		Scene 2		Scene 3			
		HH	HV	HH	HV	HH	HV	VH	VV
A	FCN16	**0.9964**	**0.9963**	**0.9957**	**0.9958**	**0.9914**	**0.9912**	**0.9912**	**0.9905**
	FCN	0.9905	0.9914	0.9907	0.9910	0.9893	0.9894	0.9897	0.9848
	M1	0.9093	0.9587	0.9830	0.9875	0.9877	0.9873	0.9883	0.9844
R	FCN16	**0.8946**	**0.8906**	**0.9344**	**0.9368**	**0.9066**	**0.9141**	**0.9168**	**0.8569**
	FCN	0.7955	0.8006	0.8531	0.8749	0.8666	0.9021	0.9114	0.7052
	M1	0.8101	0.7712	0.7189	0.7298	0.7681	0.7381	0.7766	0.6982
P	FCN16	**0.9306**	**0.9293**	**0.9601**	**0.9599**	0.9068	0.9011	0.9001	**0.9216**
	FCN	0.7226	0.7537	0.8935	0.8853	0.8694	0.8459	0.8461	0.8989
	M1	0.1472	0.2789	0.8068	0.9227	**0.9159**	**0.9356**	**0.9235**	0.8932
F1	FCN16	**0.9123**	**0.9095**	**0.9471**	**0.9482**	**0.9067**	**0.9076**	**0.9083**	**0.8881**
	FCN	0.7573	0.7765	0.8728	0.8801	0.8680	0.8731	0.8775	0.7904
	M1	0.2492	0.4097	0.7603	0.8150	0.8355	0.8252	0.8437	0.7838

**Table 5:** Time consumed for training and test.

	FCN16	FCN	M1
Training time (seconds)	4064.408	9734.3609	
Test time (seconds)	120.7649	157.4427	1834.186

**Table 6:** Index comparison between FCN16 with p equal to 0.1 and M1. A is accuracy, R is recall, P is precision, and F1 is F1 score, given in Equation (3). The better results are in bold.

		Scene 1		Scene 2		Scene 3			
		HH	HV	HH	HV	HH	HV	VH	VV
A	FCN16	**0.9926**	**0.9938**	**0.9917**	**0.9922**	**0.9907**	**0.9906**	**0.9908**	**0.9873**
	M1	0.9093	0.9587	0.9830	0.9875	0.9877	0.9873	0.9883	0.9844
R	FCN16	**0.8224**	**0.8178**	**0.8466**	**0.8615**	**0.8603**	**0.8663**	**0.8780**	**0.7463**
	M1	0.8101	0.7712	0.7189	0.7298	0.7681	0.7381	0.7766	0.6982
P	FCN16	**0.7930**	**0.8435**	**0.9267**	**0.9251**	0.9066	0.8992	0.8938	**0.9251**
	M1	0.1472	0.2789	0.8068	0.9227	**0.9159**	**0.9356**	**0.9235**	0.8932
F1	FCN16	**0.8074**	**0.8304**	**0.8848**	**0.8922**	**0.8828**	**0.8825**	**0.8858**	**0.8261**
	M1	0.2492	0.4097	0.7603	0.8150	0.8355	0.8252	0.8437	0.7838

**Table 7:** The indices of different conditions. A is accuracy, R is recall, P is precision, F1 is F1 score, given in Equation (3). The results better than FCN16 are in bold.

		Scene 1		Scene 2		Scene 3			
		HH	HV	HH	HV	HH	HV	VH	VV
A	FCN16-pre	0.9931	0.9935	0.9935	0.9938	0.9870	0.9873	0.9876	0.9805
	FCN16 + filter	0.9963	0.9962	**0.9958**	**0.9959**	0.9908	0.9905	0.9905	0.9903
	FCN16 + L2	0.9903	0.9924	0.9900	0.9908	0.9896	0.9900	0.9907	0.9839
R	FCN16-pre	0.8799	0.8451	0.9284	0.9000	0.8689	0.7428	0.7565	0.5504
	FCN16 + filter	**0.8993**	**0.8945**	**0.9479**	**0.9510**	**0.9150**	**0.9227**	**0.9253**	**0.8643**
	FCN16 + L2	0.7599	0.7351	0.7767	0.7977	0.7986	0.8122	0.8367	0.6276
P	FCN16-pre	0.7760	0.8127	0.9017	0.9315	0.8213	**0.9307**	**0.9243**	**0.9476**
	FCN + filter	0.8998	0.8988	0.9402	0.9397	0.8656	0.8543	0.8532	0.8940
	FCN + L2	0.7303	0.8384	0.9480	0.9480	**0.9366**	**0.9318**	**0.9259**	**0.9613**
F1	FCN16-pre	0.8247	0.8286	0.9149	0.9155	0.8444	0.8262	0.8320	0.6963
	FCN + filter	0.8995	0.8966	0.9440	0.9453	0.8896	0.8872	0.8878	0.8788
	FCN + L2	0.7448	0.7834	0.8538	0.8664	0.8621	0.8679	0.8790	0.7594

**Table 8:** Index comparison for FCN16 with and without pooling layer. A is accuracy, R is recall, P is precision, and F1 is F1 score, given in Equation (3). Positive Δ (in bold) means reducing pooling layer is a better choice.

		Scene 1		Scene 2		Scene 3			
		HH	HV	HH	HV	HH	HV	VH	VV
A	Simplified model	0.9945	0.9947	0.9926	0.9929	0.9896	0.9902	0.9905	0.9853
	Δ	**0.0032**	**0.0035**	**0.0014**	**0.0025**	−0.0061	−0.0056	−0.0059	−0.0110
R	Simplified model	0.7593	0.7715	0.8477	0.8560	0.8116	0.8430	0.8563	0.6858
	Δ	−0.1473	−0.1426	−0.0691	−0.0010	−0.1228	−0.0937	−0.0383	−0.2048
P	Simplified model	0.9344	0.9329	0.9497	0.9513	0.9066	0.8992	0.8938	0.9339
	Δ	**0.0276**	**0.0318**	**0.0496**	**0.0297**	−0.0375	−0.0515	−0.0271	**0.0047**
F1	Simplified model	0.8378	0.8446	0.8958	0.9011	0.8635	0.8745	0.8792	0.7909
	Δ	−0.0575	−0.0470	**0.0046**	**0.0114**	−0.0850	−0.0746	−0.0240	−0.1111
